# Design and evaluation of structural risk mitigation measures for transmission lines micro-pile foundations in mountainous region

**DOI:** 10.1371/journal.pone.0341846

**Published:** 2026-02-06

**Authors:** Xin Hu, Xiaojuan Xi, Zijun Xiang

**Affiliations:** 1 State Grid Henan Economic Research Institute, Zhengzhou, Henan Province, China; 2 College of Electrical Engineering and New Energy, China Three Gorges University, Yichang, Hubei, China; UNIPI: Universita degli Studi di Pisa, ITALY

## Abstract

Influenced by the complex geological conditions in mountainous region, micro-pile foundation for transmission line faces the risk of insufficient bearing performance. It is important to study the risk suppression measures of micro-pile foundation and its assessment method to promote the construction of transmission lines in mountainous regions. Firstly, the mechanical simulation model of pile-soil system for the micro-pile foundation is established in this paper, and the field test is carried out to verify the accuracy of the simulation model, thus the ultimate load of the micro-pile foundation is determined according to the current code requirement for maximum displacement in case of damage to the group pile foundation. Secondly, to address the subjectivity of traditional methods, an improved Likelihood-Exposure-Consequence (LEC) method is proposed. Its novelty lies in constructing a quantitative displacement-risk mathematical mapping, directly linking the physical limit state (maximum displacement) to the risk likelihood factor. Thirdly, structural risk reduction measures for the micro-pile foundation using micro-expanded pile foundation and micro-inclined pile foundation are proposed, and the ultimate load of the traditional micro straight pile foundation is used as an excitation to carry out the simulation of the bearing performance of the two improved micro-pile foundations, and the maximum displacements of the two improved micro-pile foundations are calculated. Finally, based on the proposed improved LEC method, the risk values-defined in the LEC framework as the quantitative product of Likelihood (*L*), Exposure (*E*), and Consequence (*C*)-and risk classes of the two improved micro-pile foundations are calculated and compared with the conventional micro straight pile. The results demonstrate that the proposed strategies significantly reduce the safety risk class, providing a robust, quantifiable basis for optimizing foundation designs in complex mountainous terrain.

## 1. Introduction

The entire process of mechanized construction of transmission lines in mountainous regions [1–3] includes ten key steps: project planning and feasibility studies, line design, land acquisition and approval, material transportation, foundation construction, tower assembly, line stringing, grounding, equipment installation and debugging, as well as line testing and acceptance. Among these, due to terrain and geological conditions, micro-pile foundations are commonly selected for the foundation construction stage of transmission line projects in mountainous regions [4–6]. Due to the pile-soil interaction and group pile effects [7], micro-pile foundations face the risk of insufficient bearing capacity. Therefore, proposing risk mitigation measures for micropile foundations of mountainous transmission lines and quantitatively assessing them is of great significance for promoting the mechanized transformation of transmission line construction in mountainous region.

Considerable research has been conducted both domestically and internationally regarding the bearing performance of micro-pile foundations. Liu Jinli and others, through vertical load tests on micro-pile foundations, clarified the relationship between the group pile effect and the geometric parameters of the micro-pile foundation [8]. Yan Kunfa and others, using the finite element method, analyzed the impact of the number of piles on the vertical bearing capacity of micro-pile foundations. They found that the number of piles is closely related to the settlement of micro-pile foundations, with fewer piles resulting in greater settlement. As the number of piles increases, the group pile effect becomes more pronounced [9]. M. Ebadi-Jamkhane and others also used the finite element method to study the effects of pile length and diameter on the bearing performance of micro-pile foundations. They concluded that both increasing the length and diameter of piles improves the bearing capacity and reduces displacement, though the effect of increasing pile length is more significant [10]. Tejinder Thakur and others studied the relationship between the pullout performance of micro-pile foundations and the pile embedment ratio, diameter, soil relative density, grouting pressure, grouting flow rate, and different proportions of grout materials. The results showed that the pile embedment ratio and diameter are dominant in determining the pullout performance of micro-pile foundations, while other parameters mainly influence the displacement. They suggested that the pullout bearing capacity can be improved by adjusting the geometric size of the micro-pile foundation [11].

In addition to the geometric parameter optimizations mentioned above, scholars globally have explored optimization strategies regarding pile morphology and load-bearing angles. Regarding pile morphology, some studies have proposed micro under-reamed (enlarged-base) pile schemes. By incorporating enlarged sections at key load-bearing zones to increase the pile-soil contact area, these piles enhance both end-bearing and skin friction resistance. However, most of these studies focus on ordinary foundations in flat plain areas and fail to fully account for the influence of group pile effects on the enlarged sections within complex mountainous terrain [12]. Regarding the optimization of load-bearing angles, micro-inclined piles have seen preliminary application in slope reinforcement projects due to their ability to align the pile inclination with external load directions and reduce horizontal displacement. However, research remains scattered regarding their bearing mechanisms and risk mitigation effectiveness specifically for micropile group foundations used in mountainous transmission lines [13–14]. Furthermore, some studies have proposed a “combined inclined and under-reamed” pile form. While theoretically capable of integrating the advantages of both individual optimization schemes, it imposes extremely high requirements on soil stability during actual construction. Due to the presence of the inclination angle, the stress equilibrium of the soil above the “bell face” (the transition zone between the straight shaft and the enlarged base) is easily disrupted, making it highly prone to hole collapse. For the mountainous micropile foundations investigated in this study, although such collapses are unlikely to trigger large-scale safety accidents, they directly destroy the undisturbed soil structure surrounding the pile. This leads to two critical issues: first, it obstructs subsequent pile formation; second, even if construction proceeds within the collapsed zone, the synergistic pile-soil interaction is significantly weakened. This ultimately results in substandard foundation bearing capacity that fails to meet engineering requirements. Consequently, the “combined inclined and under-reamed” pile configuration is rarely adopted in micropile foundation projects for mountainous transmission lines.

Currently, there is a lack of research specifically addressing the risk assessment of micropile foundations. Consequently, reference can be made to assessment methods employed in other sectors of power transmission and transformation engineering. At present, common methods for evaluating engineering operations include the Delphi method [15], the Analytic Hierarchy Process (AHP) [16], and the Fuzzy Comprehensive Evaluation method [17]. However, the results of these assessment methods are significantly influenced by subjective expert opinions. Furthermore, they require the collection of extensive expert feedback or scoring prior to evaluation, making it difficult to implement risk assessment directly at the micropile foundation construction site.

In comparison, the Likelihood-Exposure-Consequence (LEC) safety risk evaluation method [18–19], as a specialized tool for assessing engineering operation risks, calculates the risk value and risk level by evaluating and quantifying three factors: the likelihood of an accident, the frequency of exposure to hazardous environments, and the potential consequences. It offers the advantages of simplicity and ease of use, enabling rapid risk assessment directly at the engineering site. However, the original LEC method also suffers from reliance on empirical experience and significant subjectivity. To address this issue, existing studies in the engineering field have improved the objectivity and adaptability of the LEC method through multi-dimensional approaches. Huang [20] addressed the issues of underutilized safety big data and insufficient objectivity in safety risk evaluation for large-scale engineering construction by proposing an evaluation model based on Dempster-Shafer (D-S) evidence theory fusion and an improved LEC method. This method identifies hazard sources and quantifies risk indicators using hazard keywords and strong association rules. It then utilizes D-S evidence theory to process expert risk estimates, thereby optimizing the indicator values for personnel exposure and accident severity. Finally, a model is established by integrating these with the improved LEC method. Validated through a case study of an underground powerhouse project, the proposed method effectively enhances the accuracy and objectivity of hazard risk evaluation. Huang [21] focused on the construction safety evaluation of pumped-storage hydroelectric projects. Targeting the defects of high subjectivity and insufficient consideration of risk factors in the original LEC method, improvements were made from two perspectives: mitigating subjective influence and refining evaluation indicators. Expert credibility was introduced to attenuate individual subjective bias, while LEC parameters were modified to supplement evaluation dimensions. Application in a pumped-storage shaft project demonstrated that the improved method not only reduced subjective interference but also made indicator assignment more comprehensive, offering a reference for similar projects.

Therefore, this paper conducts pile-soil system mechanical simulation analysis of traditional micro-straight pile foundations for transmission lines in mountainous areas, determines the ultimate load of the traditional micro-straight pile foundation, and correlates the maximum displacement under this ultimate load with the maximum values of the three factors in the original LEC method. This results in the development of a displacement-risk mathematical mapping for micro-pile foundations, leading to the proposed improved LEC method for risk assessment of micro-pile foundations. On this basis, to address the risks caused by the group pile effect in traditional micro-straight pile foundations, risk mitigation measures using micro-expanded diameter piles and micro-tilted piles are proposed. The ultimate load of the micro-straight pile foundation is applied to both the micro-expanded diameter pile foundation and the micro-tilted pile foundation. The maximum displacement of the two improved micro-pile foundations is determined, and based on the improved LEC method, the risk values and risk levels of the two improved micro-pile foundations are calculated, compared with traditional micro-straight pile foundations, and the effectiveness of risk mitigation measures for the micro-expanded diameter pile foundation and micro-tilted pile foundation is evaluated.

## 2. Micro-pile foundations for transmission lines in mountainous regions and the associated risks

Due to the varied terrain and complex geological conditions in mountainous regions, which often include steep slopes, deep soil layers, unstable rock structures, and frequent geological activities (such as landslides and mudflows), traditional pile foundations for transmission line projects face numerous challenges in these areas. Firstly, traditional pile foundation construction requires the use of large mechanical equipment, which is difficult to deploy in complex terrain and can easily trigger slope instability [22–23]. Secondly, the thick overburden and complex geotechnical structures make it difficult to form boreholes, often requiring techniques such as blasting or large-diameter drilling. These methods not only result in long construction periods and high costs but can also cause environmental damage to the mountainous ecosystem. Moreover, traditional pile foundations have poor adaptability to geological activities, leading to the risk of foundation displacement or damage in areas with frequent geological events.

**Fig 1:**
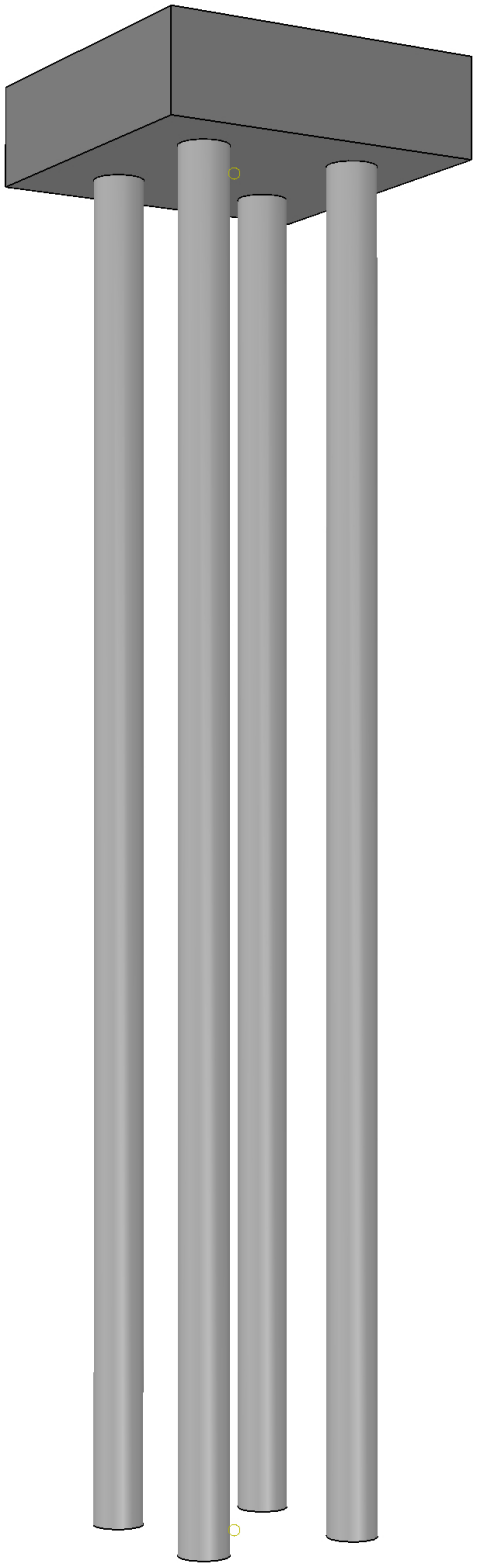
Basic Structure of a Typical Micro-Pile Foundation.

In comparison to traditional pile foundations, micro-pile foundations use techniques such as drilled grouting, jet grouting piles, or precast piles. These methods enable mechanized construction using small equipment. Additionally, through the arrangement of group piles, they ensure that the pile foundation’s bearing capacity meets the requirements for transmission tower stability while minimizing disturbances to the mountainous ecosystem. Micro-pile foundations have advantages such as small pile diameters, convenient construction, and strong adaptability [24]. Therefore, micro-piles are typically used as foundation solutions for transmission towers in mountainous transmission line projects.The basic structure of a typical micro-straight pile foundation is shown in Fig 1, consisting of an upper cap and a lower pile cluster. The cap is made of reinforced concrete, serving as a load distribution element. The piles are arranged in a rectangular array, and through the pile-soil interaction, they bear both the vertical and horizontal loads of the transmission tower. This structure not only meets the engineering mechanical requirements but also adapts to the complex terrain and geological conditions of mountainous regions, providing security for the safe and stable operation of transmission line projects in these areas.

Micro-pile foundations play a critical role in the mechanized construction of transmission lines in mountainous regions. Their application not only enhances the economic efficiency of transmission line projects in these areas but also promotes environmental protection. However, due to the complex geological conditions and harsh construction environments in mountainous regions, micro-pile foundation construction faces numerous risks. On one hand, the bearing capacity of micro-pile foundations is highly dependent on the synergistic effect of the soil between piles. When the soil strength is insufficient or disturbed, the bearing capacity is significantly reduced. On the other hand, due to the group pile effect, micro-pile foundations tend to experience uneven load distribution during load-bearing. This issue is particularly pronounced in heterogeneous layers or rock fissures, greatly increasing both design and construction difficulties. The risks faced by micro-pile foundations can, at best, affect the quality and progress of mechanized transmission line construction, or, at worst, result in safety accidents. Therefore, proposing targeted risk mitigation measures and evaluation methods for micro-pile foundations to ensure the safe and efficient mechanized construction of transmission lines in mountainous areas is of significant engineering practical importance for advancing the construction of the regional power grid.

## 3. Risk assessment method for micropile foundations of mountainous transmission lines

### 3.1. Original LEC risk assessment method

Before proposing risk mitigation measures for micropile foundations, it is necessary to seek a quantitative risk assessment method for micropile foundations.The LEC safety risk assessment method, as a tool for evaluating the hazard level of engineering operations, is characterized by its simple principles, convenience, and efficiency. It allows safety management personnel on-site to evaluate risk sources in a short period, and it is supported by explicit industry standards—specifically, the State Grid Corporation of China Enterprise Standard Q/GDW12152–2021 “Code for Safety Risk Management of Power Transmission and Transformation Engineering Construction” [25]. Therefore, it has been widely applied in the risk management of construction projects for power transmission and transformation engineering.According to the principles of the LEC method, the risk level of engineering operation accidents is determined by the risk value, which is calculated by multiplying three risk factors, namely:


D=L·E·C

In the equation: *D* represents the risk value; *L* represents the likelihood of the risk occurring; *E* represents the frequency of the risk occurrence; *C* represents the consequences when the risk occurs.

The values of the risk factors *L*, *E*, and *C* are shown in Tables 1–. After determining the values of the risk factors *L*, *E*, and *C*, the original LEC method calculates the risk value D using Equation (1). On this basis, the risk assessment of engineering operations is performed according to the relationship between risk values and risk levels shown in Table 4. However, the original LEC method still relies on the experience and judgment of on-site safety management personnel when determining the values of risk factors *L*, *E*, and C. Given that the LEC method in the aforementioned standards primarily focuses on general safety risks during the construction phase, it is difficult to directly assess the structural safety risk arising from insufficient bearing capacity of micropile foundations for transmission lines in mountainous areas. Therefore, the original LEC method needs to be improved to achieve objective valuations for the risk factors *L*, *E*, and *C*, thereby enhancing the validity of the evaluation results. It should be noted that the optimization of the LEC method in this paper is carried out entirely within the standard framework. We have not altered the evaluation logic of *L*, *E*, and C or the risk level thresholds specified in the standard. Instead, specific to the research characteristics of micropile foundations, we have supplemented a dimension linking the mechanical performance of the pile-soil system to risk. Specifically, through mechanical simulation of the pile-soil system and field tests, we obtain the maximum displacement under ultimate loads before and after foundation improvements. This displacement is then correlated with the maximum value of risk factors in the standard LEC method using an equal-ratio scaling, thereby constructing a displacement-risk mathematical mapping. This achieves a quantitative assessment of the structural safety risk of micropile foundations, ensuring that the improved method not only complies with industry standards but also meets the specific needs of the research scenario.

**Table 1:** Value Table for Risk Factor *L.*

Value	Likelihood of risk occurrence
10	Very likely
6	Likely
3	Infrequent likelihood
1	Unlikely, completely unexpected
0.5	Highly unlikely, but conceivable
0.2	Extremely unlikely
0.1	Practically impossible

**Table 2:** Value Table for Risk Factor *E.*

Value	Frequency of Risk Occurrence
10	Continuous
6	Once a day
3	Once a week
2	Once a month
1	A few times a year
0.5	Very rare

**Table 3:** Value Table for Risk Factor *C.*

Value	Consequences of Risk Occurrence
40	Disaster, nearly unbearable loss
15	Very serious, major loss
7	Significant loss
3	Major loss
1	Moderate loss
0.5	Minor loss

**Table 4:** Relationship between Risk Value *D* and Risk Level.

Risk Value *D*	Risk Severity	Risk Level
*D* ≥ 320	Extreme risk, cannot continue operation	1
160 ≤ *D < *320	High risk, strict supervision required	2
70 ≤ *D < *160	Significant risk, dedicated supervision required	3
20 ≤ *D < *70	Moderate risk, attention required	4
*D < *20	Slight risk, acceptable	5

### 3.2. Improved LEC risk assessment method

For micro-pile foundations, the better the bearing performance, the lower the likelihood of risk occurrence; that is, the smaller the value of the risk factor *L*. It should be noted that, regardless of the measures taken to improve the ultimate bearing performance of the micro-pile foundation, the risk mitigation measures proposed in this paper target only the risk changes of the same type of foundation before and after structural form improvements. It involves no cross-category evaluation of different foundation types, thereby ensuring a unified risk baseline for the evaluation objects. Since the improvement measures only optimize the pile morphology to enhance bearing capacity, they do not alter core risk-triggering conditions such as the geological environment or engineering loads. Furthermore, the same type of foundation performs consistent functions in transmission lines and has consistent connection modes with the main structure; thus, the severity of failure consequences remains stable and is unaffected by differences in construction difficulty. Consequently, the frequency of risk occurrence and the consequences can be considered approximately unchanged; that is, the values of risk factors *E* and *C* remain constant. In this paper, they are set to 1 to ensure that the evaluation results solely reflect the risk reduction effect of foundation performance improvements on bearing failure risk, avoiding interference from irrelevant variables. It is important to note that setting *E* and *C* to 1 is a simplification valid specifically for the relative comparison of foundation alternatives under identical environmental and operational conditions. For other engineering scenarios involving different transmission line importance classes, varying seismicity levels, or distinct slope stability conditions, the values of *E* and *C* must be adjusted according to the specific project context.

It should be clarified that the linear relationship established here is a definition of the risk metric, rather than a representation of the physical non-linear mechanical behavior of the pile-soil system. While the geotechnical response near failure is indeed non-linear, the risk index is defined to scale linearly from a stable state (zero displacement) to the ultimate limit state (failure displacement). This linear mapping serves as a robust and neutral indexing strategy to quantify the ‘degree of safety’ in the absence of specific probability distribution data.

**Fig 2:**
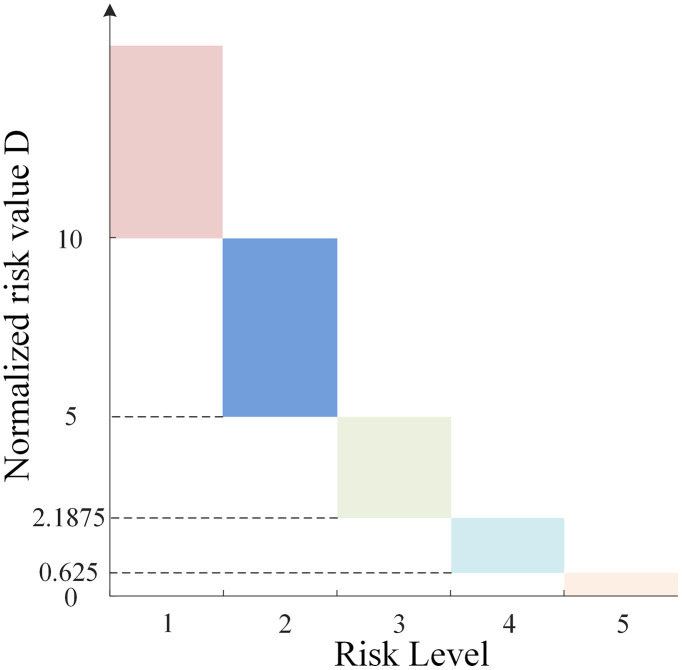
Correspondence between normalized risk value and risk level.

Therefore, the risk value assessment for micro-pile foundations of mountainous transmission lines is determined by the value of risk factor *L*. Since there is a difference in magnitude between the value of the risk factor *L* and the risk value *D*, making risk level classification difficult, it is necessary to normalize the risk value *D* based on the value of the risk factor *L*. The specific method involves calculating the multiplier relationship between the minimum risk value *D* at the highest risk level and the maximum value of the risk factor *L*, and then applying this equal multiplier to normalize risk values across different risk levels. The normalization factor is derived from the ratio of the minimum risk value *D* required for the highest risk level (Level 1) to the maximum possible value of the likelihood factor *L*. According to Tables 1 and 4, the minimum *D* for Level 1 is 320, and the maximum *L* is 10. Therefore, the scaling multiplier is calculated as 320/10 = 32. This ensures that when the likelihood is at its maximum (approaching failure), the resulting risk value aligns with the threshold for “Extreme Risk”.The normalization factor of 32 used in this study is case-specific and derived from the ultimate displacement threshold observed under the specific geological conditions and pile dimensions of this project. It is not intended to be a universal constant. For engineering applications in different geological contexts or with different foundation types, this normalization factor should be recalibrated. Engineers should determine the factor based on the specific allowable deformation limits or ultimate load criteria relevant to their project requirements. After applying this equal-ratio normalization, the relationship between the risk value and the risk level is shown in Fig 2.

The bearing capacity of micro-pile foundations is typically assessed using pile-soil displacement under the ultimate load. Therefore, it is necessary to correlate pile-soil displacement with the normalized risk value. To this end, this paper first establishes a mechanical simulation model of the pile-soil system for the micro-pile foundation and determines the ultimate load of the micro-pile foundation according to the maximum displacement requirements for group pile foundations’ failure under current standards. Then, the maximum displacement under this ultimate load is matched with the minimum risk value corresponding to the level 1 risk, and a linear mathematical mapping of displacement-risk for the micro-pile foundation is constructed, as shown in Equations (2)-(3). This approach enables the improvement of the LEC method for risk assessment of micro-pile foundations.


$$D_{i}=\frac{X}{X_{\max}}D_{1}$$

$$\begin{array}{c} G=\left\{ \begin{array}{c} 1\;X\geq X_{\max}\\ 2\;X_{\max}\cdot \frac{D_{2}}{D_{1}}\leq X<X_{\max}\\ 3\;X_{\max}\cdot \frac{D_{3}}{D_{1}}\leq X<X_{\max}\cdot \frac{D_{2}}{D_{1}}\\ 4\;X_{\max}\cdot \frac{D_{4}}{D_{1}}\leq X<X_{\max}\cdot \frac{D_{3}}{D_{1}}\\ 5\;X\geq X_{\max}\cdot \frac{D_{4}}{D_{1}} \end{array}\right. \end{array}$$

In the equation: *D*_*i*_ represents the risk value calculated using the improved LEC method; *G* represents the risk level; *X* represents the pile-soil displacement of the micro-pile foundation; *X*_*max*_ represents the pile-soil displacement of the micro-pile foundation under the ultimate load; *D*_*1*_ to *D*_*4*_ are the risk threshold values corresponding to each risk level. According to Fig 2, *D*_*1*_ = 10, *D*_*2*_ = 5, *D*_*3*_ = 2.1875, and *D*_*4*_ = 0.625.

## 4. Design and evaluation of risk mitigation measures for micro-pile foundations

### 4.1. Risk mitigation measures

#### 4.1.1. Enlarged diameter pile foundation.

**Fig 3:**
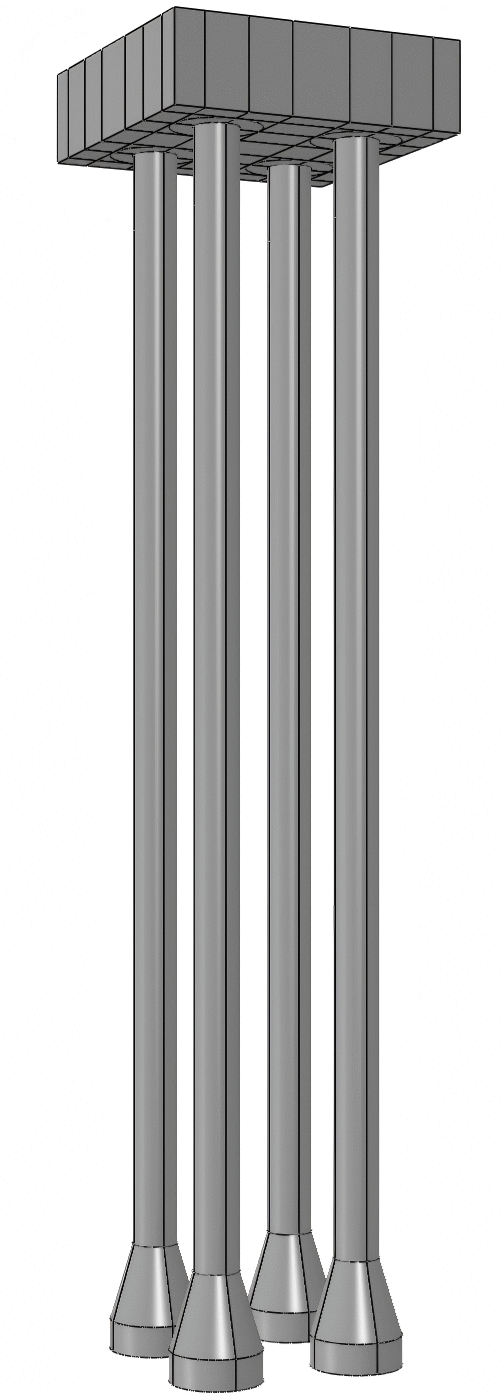
Basic Structure of Enlarged Diameter Pile Foundation.

Compared to a straight pile foundation, the characteristic of an enlarged diameter pile foundation is the presence of a section with a diameter change on the pile body, known as the enlarged diameter head, whose basic structure is shown in Fig 3. The enlarged diameter head can significantly reduce stress concentration without disrupting the soil’s static state. The main advantage of the enlarged diameter pile foundation over the straight pile foundation is that the presence of the enlarged diameter head increases the foundation’s ultimate uplift load. The ultimate uplift load of an enlarged diameter pile foundation typically consists of two components: one is the side friction resistance carried by the pile body, which is more common in scenarios with higher soil strength and better pile-to-soil friction performance, and this forms the main component of the ultimate uplift load of the enlarged diameter pile foundation; the second is the anchoring effect from the enlarged diameter head, which usually occurs when the enlarged diameter head is anchored in soil layers with high bearing capacity.

#### 4.1.2. Inclined pile foundation.

**Fig 4:**
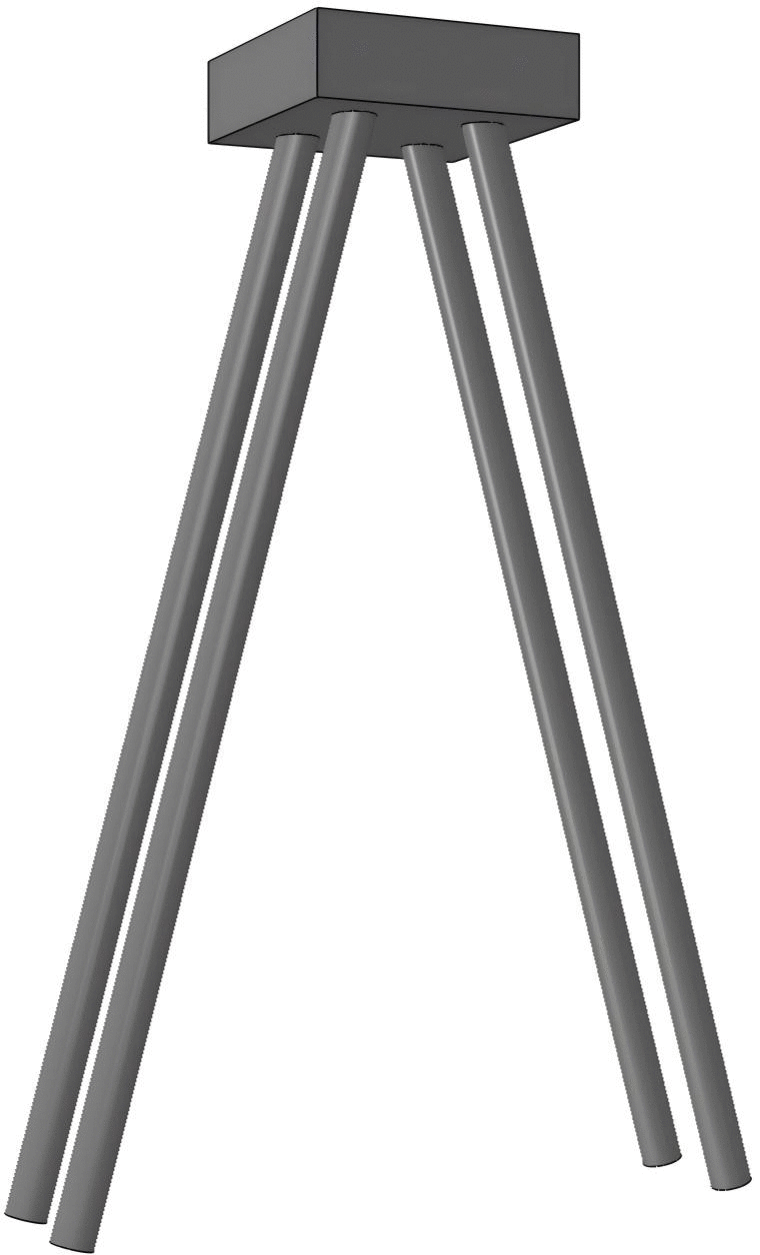
Basic Structure of Inclined Pile Foundation.

Compared to a straight pile foundation, the characteristic of an inclined pile foundation is that some or all of the piles are arranged in an inclined manner, with the basic structure shown in Fig 4.The main advantage of an inclined pile foundation over a straight pile foundation is that the inclined piles can significantly increase the foundation’s ultimate horizontal load capacity. Based on the relationship between the direction of the horizontal load and the inclination of the pile, inclined pile foundations can be classified into positive inclined piles and negative inclined piles. Piles inclined in the same direction as the horizontal load are typically called positive inclined piles, while those inclined in the opposite direction are called negative inclined piles. When resisting horizontal loads, the horizontal bearing capacity of positive inclined piles is superior to that of negative inclined piles. Positive inclined piles can distribute and transfer the horizontal load, thus preventing localized stress concentrations in the pile. In addition, the bending deformation of positive inclined piles increases significantly with the inclination angle, indicating that their structural stiffness improves as the angle of inclination increases, whereas the opposite is true for negative inclined piles. In practical engineering, as the foundation may be subjected to horizontal loads from different directions, inclined pile foundations are typically arranged symmetrically with a set of positive inclined piles and a set of negative inclined piles to increase the ultimate horizontal load capacity of the inclined foundation.

### 4.2. Risk mitigation evaluation

#### 4.2.1. Simulation model and experimental validation.

**Fig 5:**
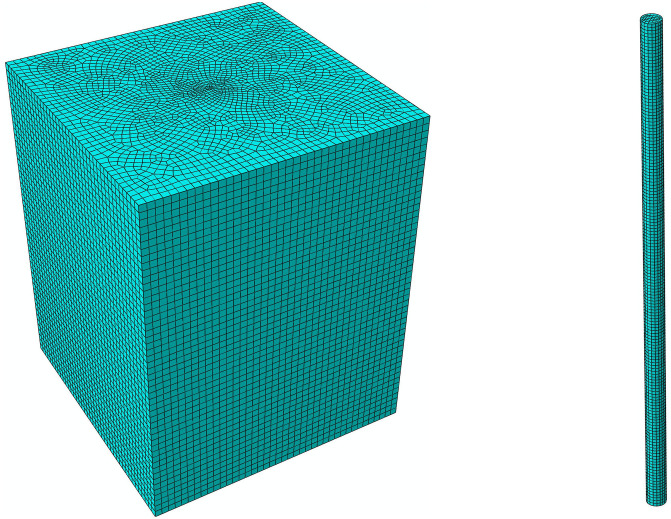
Soil and experimental micro pile model.

While compressive and horizontal bearing capacities are essential for overall stability, uplift bearing capacity is typically the controlling design factor for transmission line foundations in mountainous terrain due to the significant overturning moments caused by wind and ice loads. Therefore, this study prioritizes the analytical validation of the ultimate uplift capacity to verify the model’s reliability, while compressive and horizontal behaviors are assessed via the validated numerical model. This study employs the simulation software Abaqus to analyze the mechanical characteristics of the pile-soil system for a vertical micropile. The accuracy of the resulting simulation model and its methodology is then validated through corresponding physical experiments.The pile has a diameter of 0.3 m and a length of 8 m, situated within a soil domain measuring 12m × 12m × 15m.To maintain consistency with the experimental conditions, in which ancillary foundation structures such as a pile cap were not constructed, these elements were also excluded from the simulation model.The detailed simulation model is illustrated in Fig 5.

In the simulation model, the micropile body is composed of C25 concrete, with mechanical parameters determined in accordance with GB 50010−2010 Code for Design of Concrete Structures [26]. Specifically, the elastic modulus is taken as 2.8 × 10^4^ MPa, Poisson’s ratio as 0.2, and the standard axial compressive strength as 25 MPa. These parameters are consistent with the actual mechanical performance test results of the C25 plain concrete used in the experiment. The pile-soil interface adopts the Mohr-Coulomb model. The interface friction coefficient is set to 0.45, based on the characteristics of the soil layers at the test site (Quaternary silty gravel layer and silty sand-gravel layer) and data from similar simulation studies of micropile foundations in mountainous areas. The normal contact stiffness coefficient is derived as 1.2 × 10^8^N/m^3^ based on the soil compressibility modulus (taken as 22 MPa for silty sand-gravel), while the tangential contact stiffness coefficient is set to 0.5 times the normal contact stiffness coefficient (i.e., 6.0 × 10^7^N/m^3^).

To further validate the reliability of the numerical model, the ultimate uplift bearing capacity of the standard micro-straight pile was calculated using the theoretical limit state formulas specified in the Technical Code for Building Pile Foundations (JGJ 94–2008). The theoretical ultimate uplift capacity ($R_{uk}$) is calculated as follows:


$$R_{uk}=\sum q_{sik}l_{i}u_{i}+G_{p}$$

Where $q_{sik}$ is the standard value of the ultimate skin friction resistance of soil layer i, $l_{i}$ is the thickness of soil layer i, $u_{i}$ is the perimeter of the pile in soil layer i, and $G_{p}$ is the self-weight of the pile.

**Fig 6:**
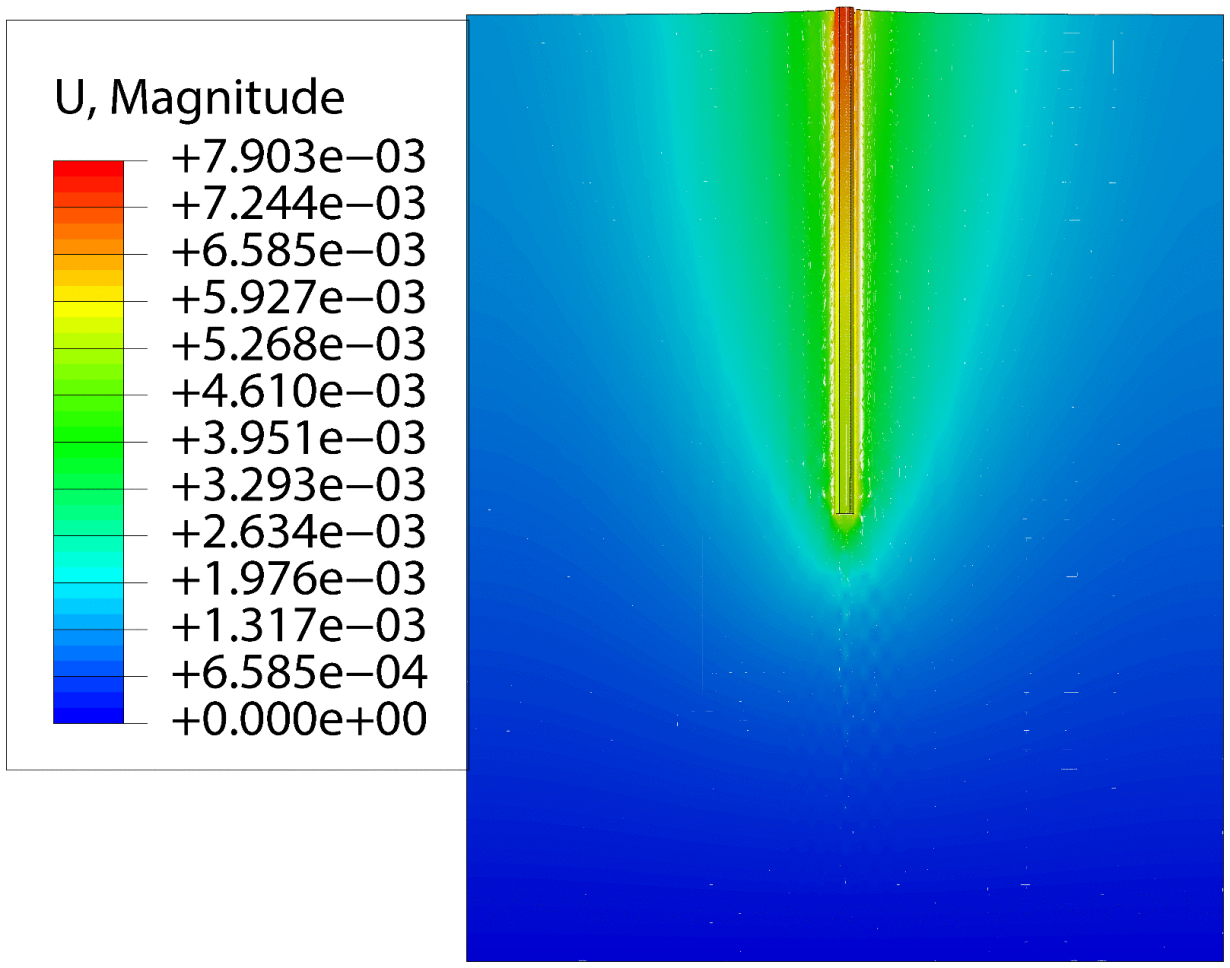
Displacement simulation results of vertical pile under ultimate uplift load.

The ultimate load was determined using an iterative load application strategy. Initially, a small load was applied, and the resulting displacement of the pile-soil system was compared against the limit specified by the relevant code. The load was then incrementally increased in subsequent simulations until the maximum displacement reached this limit. The load at this stage was identified as the ultimate load of the micropile foundation. The displacement results for the vertical micropile and its surrounding soil under the ultimate uplift load are presented in Fig 6. The ultimate uplift load was determined to be 325 kN, corresponding to a pile-top displacement of 31.03 mm.Under this ultimate load, the pile body exhibited a tendency to pull out from the soil, while the surrounding soil mass displayed a concurrent upward movement. This behavior, which is characteristic of a ‘friction pile,’ was consistent with the experimental observations, thus validating that the simulation model accurately reproduced the mechanical behavior from the physical tests.

The ultimate uplift load experiment on the vertical micropile was conducted at a test site in the mountainous region of western Zhumadian City, Henan Province. The site, situated at an elevation of approximately 382 meters, features significant topographic relief, and its characteristic mountainous geomorphology contributes to complex soil stratigraphy and geological conditions. No specific permits were required for the described field studies as the site is not a protected area. The soil profile in this region consists of alternating layers of silt and gravelly soil, with some tower foundations established directly within these strata.Through field exploration and laboratory geotechnical tests, it was determined that the lithology of the local stratum is relatively simple, primarily comprising two main layers: a Quaternary silty gravel layer and a silty sand-gravel layer.Based on water level observations from three on-site exploration boreholes (conducted one week prior to the test with 72 hours of continuous monitoring), the groundwater table depth at the test site remained stable between 10.5 and 12.0 m. This is significantly lower than the 8 m length of the micro-straight pile used in this test, indicating that the entire pile body is located within the vadose zone (unsaturated soil layers) above the groundwater table. Meanwhile, combined with borehole water injection tests, the permeability coefficient of the Quaternary silty gravel layer at the site was measured at 1.2 × 10^-3 cm^/s, and that of the silty sand-gravel layer was 8.5 × 10^-4 cm^/s. The overall permeability of the soil layers is favorable, allowing small amounts of pore water to infiltrate downward rapidly, thereby preventing the formation of significant water accumulation or saturated zones around the pile. Moisture content test results for soil samples around the pile show that within the 0 ~ 8.0 m depth range of the pile shaft, the soil moisture content generally remains stable between 19.89% and 22.18%, with no obvious saturated sections. Under this moisture content range, both the inter-particle friction and soil cohesion of the silty gravel and silty sand-gravel layers remain in a stable state, without significant softening or strength degradation caused by moisture. In summary, given that the groundwater depth is far greater than the pile length, and the soil layers exhibit good permeability and low moisture content, groundwater has minimal influence on the pile-soil interface friction characteristics and the strength of the soil surrounding the micro-straight pile in this test Table 5.

**Fig 7:**
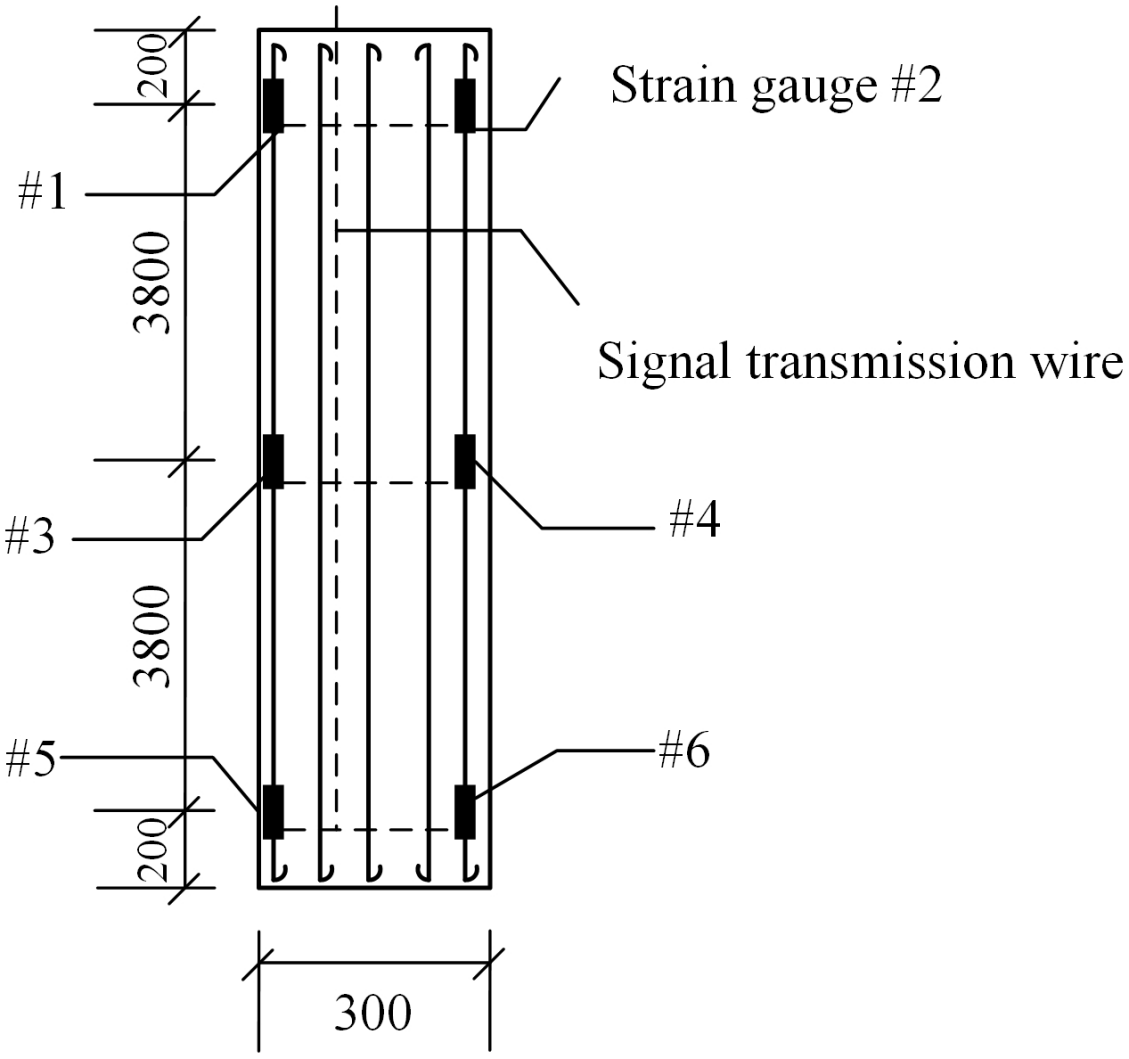
Strain gauge layout diagram.

**Fig 8:**
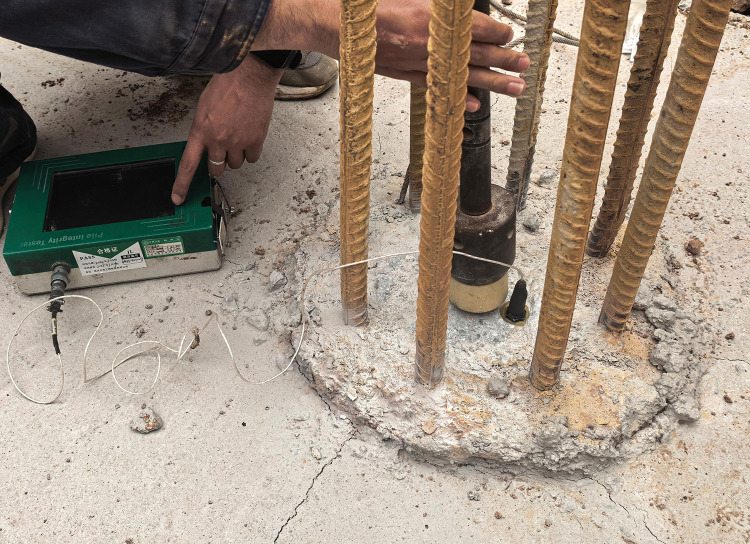
Pile integrity testing.

During the experiment, six rebar strain gauges were installed at various embedment depths—specifically, 200 mm below the pile top, at the pile midpoint, and 200 mm above the pile toe—to ensure precise monitoring of the pile body’s response at its top, middle, and bottom sections. Additionally, a pair of rebar strain gauges was installed within each soil layer penetrated by the pile. The arrangement of the strain gauges is illustrated in Fig 7. To further enhance the test’s accuracy, a pile testing instrument was used to monitor deformation, displacement, and stress distribution. Prior to the experiment, this instrument was also used to perform a pile integrity test (as shown in Fig 8) to ensure that casting imperfections would not lead to data deviations.

The steel reinforcement strain gauge used in this test is model BX120–3AA, featuring a sensitivity coefficient of 2.00 ± 0.01, a gauge length of 3 mm, and an insulation resistance of ≥5000MΩ at ambient temperature, which meets the precision requirements for pile strain monitoring. The pile testing instrument employed is the PIT-VV model pile integrity tester. Specialized calibration was completed within 15 days prior to the test. In addition to covering displacement measurement accuracy, stress signal acquisition accuracy, and data transmission stability, the calibration items also included two key indicators: the instrument’s natural frequency response and the linearity of signal gain. Specifically, the calibration confirmed that the displacement measurement error is ≤±0.1 mm, the stress signal acquisition error is ≤±2%, and the data transmission latency is ≤ 5ms. All indicators comply with the Grade I accuracy requirements specified in JJF 1337–2012 Calibration Specification for Pile Dynamic Measuring Instruments [27], with the calibration certificate number 3081C. Crucially, the calibration certificate confirms the instrument’s accuracy, with its Expanded Uncertainty directly corresponding to the 95% confidence level for all measurements. Following calibration, on-site verification was conducted on the instrument’s four signal acquisition channels under both no-load and simulated loading conditions. The deviation between the simulated load values and the instrument display values was controlled within 1%, confirming that the instrument’s calibration status was valid.

**Fig 9:**
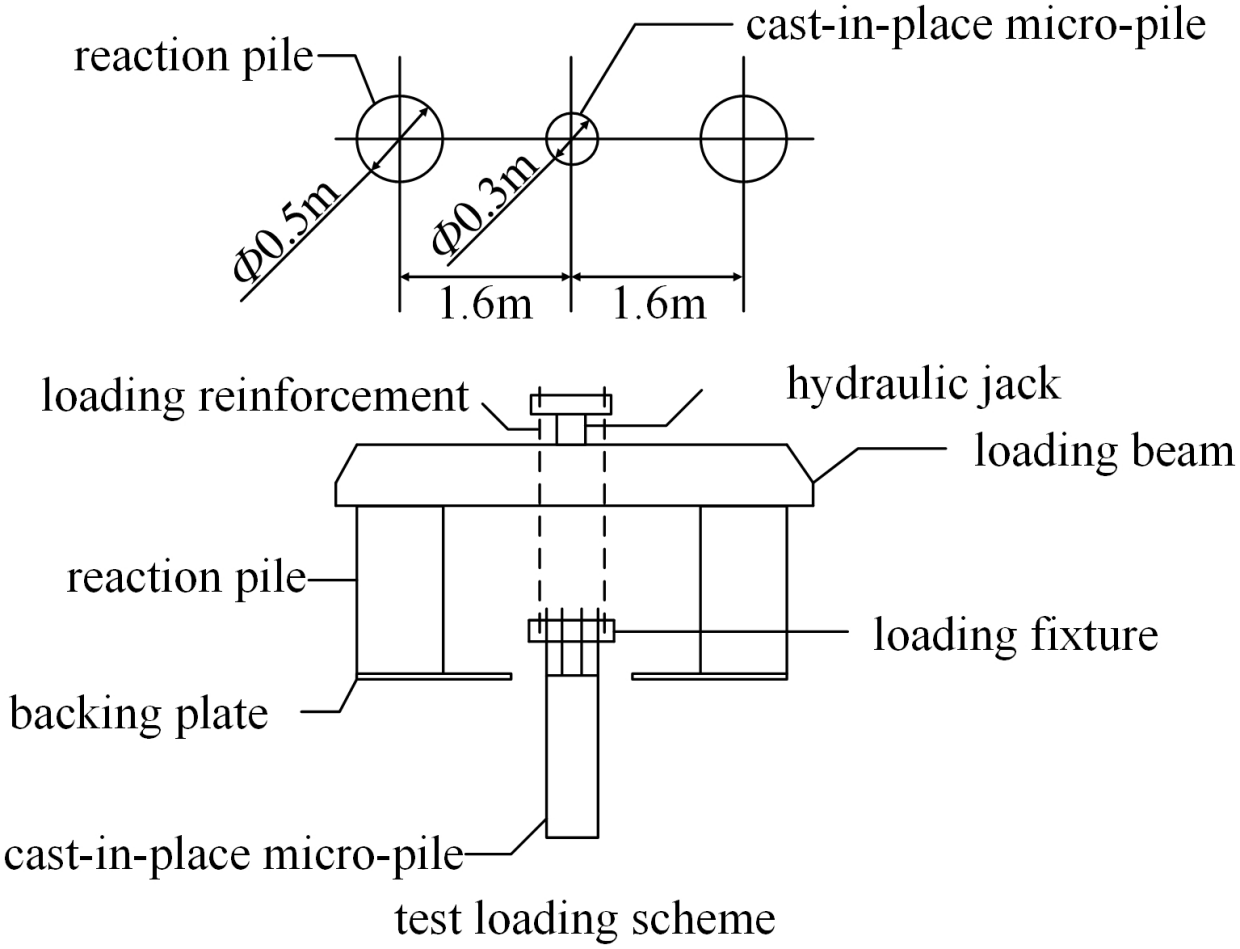
Experimental loading scheme.

The loading system used for this ultimate uplift test is depicted in Fig 9. This system consisted of a hydraulic jack to provide the uplift force, while a reaction frame—composed of a loading beam, reaction piles, and bearing plates—was established. A rigid connection to the cast-in-place micropile was achieved using two sets of loading clamps and reinforcing bars.

**Fig 10:**
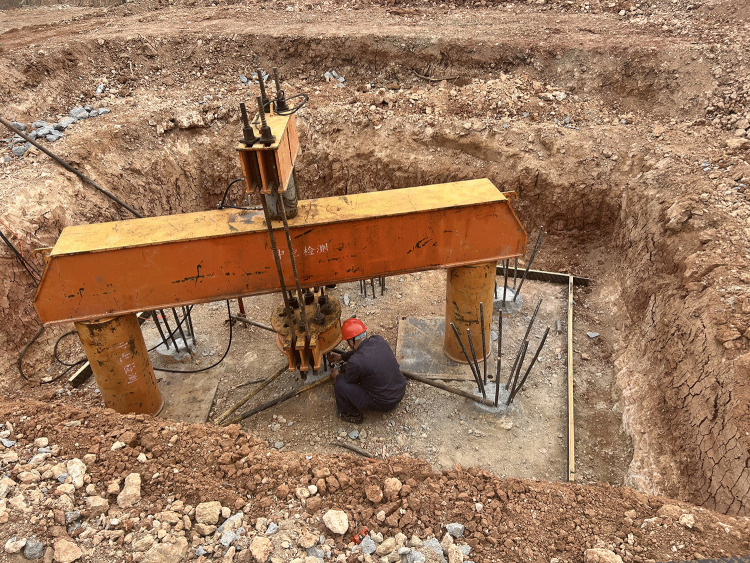
Photograph of the field experiment.

According to the Technical Specifications for Building Foundation Pile Testing (JGJ106–2014) [28], a pile foundation is considered to have failed when its pile-top displacement exceeds 30 mm under compressive or uplift loads, or when it exceeds 10 mm under horizontal loads.In this ultimate uplift load test on the vertical micropile, a staged loading strategy was adopted, with each increment being 50 kN. The experimental site is shown in Fig 10.

**Fig 11:**
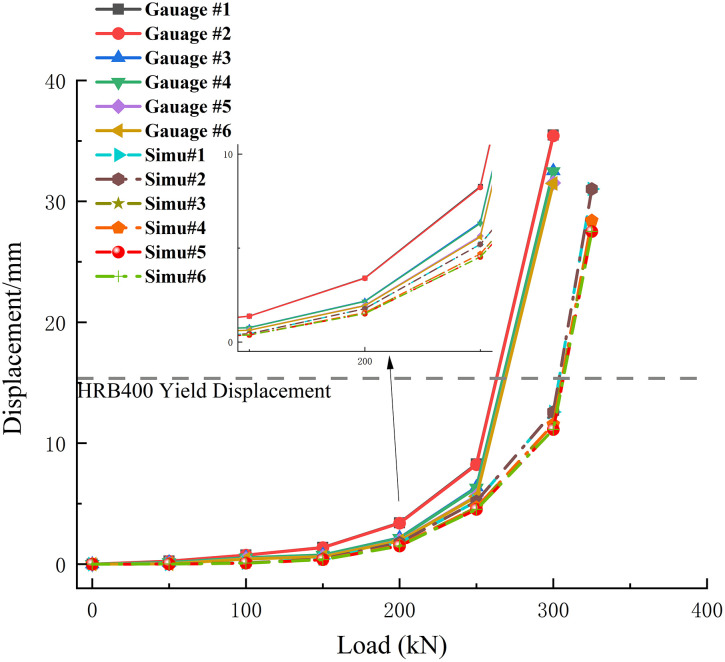
Comparison of experimental and simulated displacements under different load levels.

Fig 11 illustrates the displacement profiles corresponding to different load levels for both the experimental test and the numerical simulation, where the experimental displacements were derived from the strain gauge measurements. As shown in the figure, the strain readings from gauges located at the same vertical level in the experiment exhibit minimal variation, while the simulation results at these corresponding positions are identical due to the highly symmetrical conditions.

In the experiment, the displacements remained relatively small when the load was below 250 kN, indicating that the foundation had not yet reached its ultimate uplift state. However, as the load reached 300 kN, the displacement increased sharply and exceeded the value corresponding to the yield strain of HRB400 steel. This suggests that the reinforcement had failed; at this point, the displacement at the top of the foundation reached 33.15 mm. The simulation exhibited a similar trend regarding the load response. When the simulated load reached 325 kN, the displacements at the locations corresponding to the embedded strain gauges also increased abruptly, signifying that the foundation had reached its ultimate uplift state.

The calculated theoretical ultimate uplift capacity is 257.96 kN, whereas the experimental ultimate capacity obtained from the test is 300 kN. The relative deviation between the theoretical and experimental values is 14.01%. This discrepancy is primarily attributed to the simplifications regarding the pile-soil interface behavior in the theoretical model. Specifically, the theoretical formula typically assumes a uniform friction coefficient, whereas the surface roughness of the actual cast-in-place concrete pile induces significant mechanical interlocking with the surrounding soil. Additionally, the construction process may have increased the lateral confinement of the soil, resulting in a higher actual radial stress acting on the pile surface than the value assumed in the calculation. These factors collectively mobilized higher skin friction in the field test compared to the theoretical prediction.

The test results indicated that the ultimate uplift load for the vertical micropile foundation was 300 kN, corresponding to a maximum pile-top displacement of 33.15 mm.Compared to the measured value of the ultimate uplift load, the simulation result had an error of 8.34%. The reasons for this discrepancy are as follows:

1) In the mechanical simulation, the pile-soil interface was modeled by assigning a specific stiffness to the contact elements, and this stiffness was assumed to be constant, leading to a comparatively higher simulated load capacity. In actual conditions, however, the contact stiffness at the pile-soil interface exhibits nonlinear behavior, gradually decreasing as the load increases due to soil heave.
2) During the field test, the processes of excavation and casting for both the reaction piles and the test pile inevitably introduced a degree of soil disturbance and construction error, which in turn affected the experimental results.
3) In the field experiment, the connection between the loading bars and the clamps was not perfectly rigid, resulting in some loss during the transmission of the applied load.

Despite these discrepancies, the small error margin between the simulated and measured values indicates that the results are acceptable for practical engineering purposes. The strong agreement between the simulated mechanical behavior and the experimental data confirms that the proposed model and methodology are suitable for the risk mitigation assessment of micropile foundations.

#### 4.2.2. Mechanical properties of micro-pile foundation.

**Fig 12:**
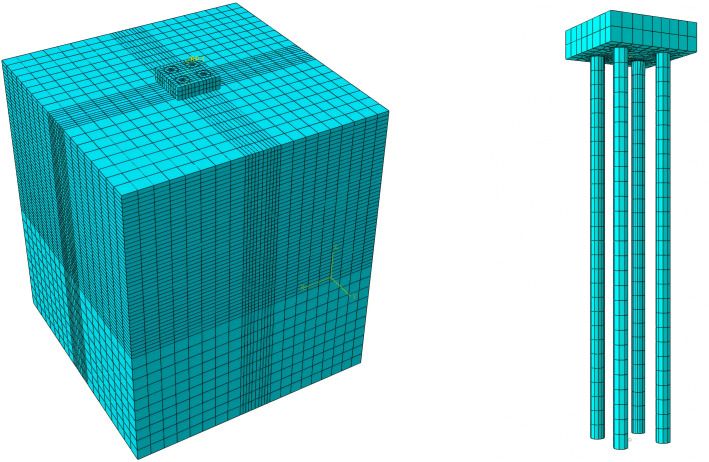
Mechanical Simulation Model of Pile-Soil System for Micro-Pile Foundation.

To verify the effectiveness of the proposed risk mitigation measures for micro-pile foundations, a pile-soil system mechanical simulation model, as shown in Fig 11, is established. Simulation analysis of the mechanical properties of the micro-pile foundation is conducted, and based on this, the risk level evaluation is carried out using the risk assessment method in Section 2 of this paper.In the pile-soil system mechanical simulation model shown in Fig 12, the micro-pile foundation is a 2 × 2 square pile group structure with a pile diameter of 0.3m, a pile length of 8m, and a pile spacing of 1m. The cap dimensions are 2m × 2m × 0.6m, and the soil dimensions are 12m × 12m × 15m.

**Fig 13:**
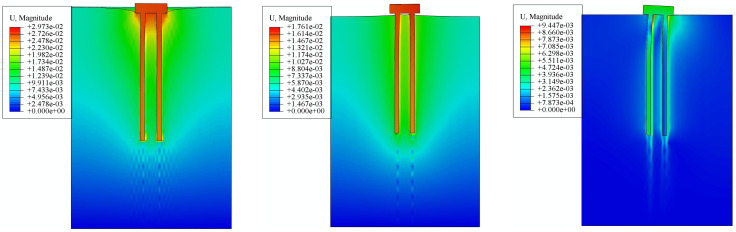
Displacement Results of Straight Pile Foundation under Ultimate Loads. (a) Displacement under Ultimate Compressive Load. (b) Displacement under Ultimate Uplift Load. (c) Displacement under Ultimate Horizontal Load.

To verify the effectiveness of the proposed risk mitigation measures, the mechanical properties of the micro-straight pile foundation are first analyzed.Through pile-soil system mechanical property simulation, the ultimate compressive load, ultimate uplift load, and ultimate horizontal load of the micro-straight pile foundation are determined to be 2800kN, 1080kN, and 620kN, respectively.The displacement simulation results of the micro-straight pile foundation under ultimate loads are shown in Fig 13. Fig 13(a) shows the displacement under the ultimate compressive load, Fig 13(b) shows the displacement under the ultimate uplift load, and Fig 13(c) shows the displacement under the ultimate horizontal load (direction to the right).From the results shown in Fig 13(a), it can be seen that under the ultimate compressive load, the displacement of the straight pile foundation and the surrounding soil mainly occur beneath the cap and the lower part of the pile. The soil displacement distribution shows that the closer the soil is to the straight pile foundation, the larger the displacement.The soil around the cap shows significant deformation due to the compressive load, with the maximum displacement reaching 29.73 mm.From the results shown in Fig 13(b), it can be seen that under the ultimate uplift load, the straight pile foundation exhibits a clear tendency to detach from the surrounding soil, indicating that the foundation has reached the ultimate uplift state.The trend of displacement magnitude in the surrounding soil is similar to that observed under the ultimate compressive load simulation, with the difference being that the displacement direction is opposite to that under the compressive load.The deformation of the straight pile foundation and the surrounding soil under the uplift load exhibits typical friction pile behavior.The soil around the cap shows deformation due to the uplift load, with the maximum displacement reaching 31.47 mm.From the results shown in Fig 13(c), it can be seen that under the ultimate horizontal load, the cap tends to move horizontally due to the horizontal forces acting on its upper surface. The pile group is hindered by the soil, and the soil can only deform slightly through compression, resulting in small deformation corresponding to the cap’s movement trend. The cap and pile group are tightly connected, thus the entire straight pile foundation exhibits typical shear force-induced deformation.In the vertical direction, the deformation of the surrounding soil of the straight pile foundation shows a unilateral “uplift” and “compression” pattern. In the horizontal direction, the soil displacement in the direction opposite to the applied force is smaller, while the soil displacement in the direction of the applied force is larger, with the maximum soil displacement being 9.45 mm.

#### 4.2.2. Mechanical properties and risk assessment of micro under-reamed pile.

**Fig 14:**
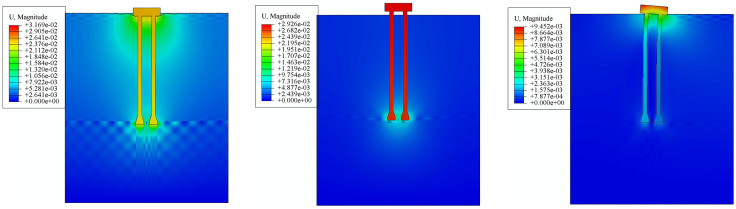
Displacement Results of Enlarged Diameter Pile Foundation under Ultimate Loads. (a) Displacement under. Ultimate Compressive Load. (b) Displacement under Ultimate Uplift Load. (c) Displacement under Ultimate Horizontal Load.

Based on the simulation model shown in Fig 5, the tail end of the micro-pile foundation’s pile body is modified to an enlarged diameter head (the enlargement factor chosen for this simulation is 2), thereby establishing a pile-soil system mechanical property simulation model for the micro-enlarged diameter pile foundation.Through the pile-soil system mechanical property simulation, the ultimate compressive load, ultimate uplift load, and ultimate horizontal load of the micro-enlarged diameter pile foundation are determined to be 5400kN, 2300kN, and 660kN, respectively.Compared to the micro-straight pile foundation, the three ultimate loads of the micro-enlarged diameter pile foundation are increased by 92.86%, 112.96%, and 6.45%, respectively. It can be observed that the micro-enlarged diameter pile primarily improves its resistance to uplift load.The displacement simulation results of the micro-enlarged diameter pile foundation under ultimate loads are shown in Fig 14. Fig 14(a) shows the displacement under the ultimate compressive load, Fig 14(b) shows the displacement under the ultimate uplift load, and Fig 14(c) shows the displacement under the ultimate horizontal load (direction to the right).

**Fig 15:**
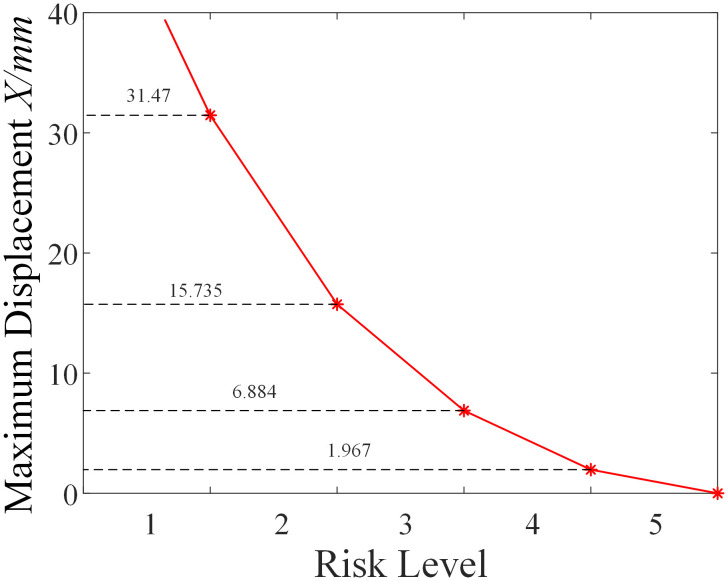
Relationship Curve between Maximum Displacement and Risk Level for Micro Under-reamed Pile.

From the results shown in Fig 14(a), it can be seen that under the ultimate compressive load, the displacement of the micro-enlarged diameter pile foundation and the deformation of the surrounding soil mainly occur beneath the cap and the enlarged diameter head. The closer the soil is to the pile group, the larger the displacement, and the entire micro-enlarged diameter pile foundation exhibits “friction pile” characteristics.Additionally, significant deformation of the soil around the cap and enlarged diameter head occurs due to the compressive load. From the displacement field shown in Fig 14(b), distinct differences are observed in the failure mechanism of the micro-enlarged diameter pile compared to the straight pile. Under the ultimate uplift load, the deformation of the surrounding soil is concentrated above the enlarged head, forming a clear “soil arching” phenomenon. This indicates a fundamental shift in the load transfer mechanism: the enlarged head acts as a mechanical anchor, mobilizing the shear strength of the overlying soil cone rather than relying solely on skin friction along the pile shaft. The “bridge arch” pattern suggests that the stress is effectively redistributed into the surrounding soil, thereby significantly enhancing the uplift resistance through end-bearing anchorage. From the results shown in Fig 14(c), it can be observed that under the ultimate horizontal load, the displacement and soil deformation of the micro-enlarged diameter pile foundation are similar to those of the micro-straight pile foundation. The deformation of “uplift” on one side and “compression” on the other side is amplified due to the effect of the enlarged diameter head, which is similar to the effect seen in compressive and uplift loads.Furthermore, the risk mitigation effect of the micro-enlarged diameter pile foundation is evaluated.The specific approach is to first assume that the micro-straight pile foundation carries its ultimate uplift load, at which point the micro-straight pile foundation faces a risk of failure. According to Fig 2, the corresponding risk value is 10, and the risk level is Level 1.Then, the ultimate uplift load of the micro-straight pile foundation is applied to the micro-enlarged diameter pile foundation. Through pile-soil system mechanical property simulation, the maximum displacement of the micro-enlarged diameter pile foundation is found to be 13.44 mm.Finally, based on the improved LEC method proposed in Section 2 of this paper, the risk value of the micro-enlarged diameter pile foundation is calculated to be 4.27. The relationship curve between maximum displacement and risk level for the micro-enlarged diameter pile foundation is shown in Fig 15.From Fig 15, it can be seen that the risk level of the micro-enlarged diameter pile foundation at this point is Level 2.Compared to the traditional micro-straight pile foundation, the risk value of the proposed micro-enlarged diameter pile foundation decreases by 57.30%, and the risk level decreases from Level 1 to Level 2, achieving risk mitigation.

Furthermore, by changing the enlargement factor, the variation of the ultimate uplift load of the micro-enlarged diameter pile foundation with respect to the enlargement factor is analyzed.According to relevant studies [29], for micropile foundations subjected to compressive loads, an enlargement ratio of 2.5 to 3.0 is typically recommended to achieve optimal bearing performance. However, the micro under-reamed pile foundations investigated in this study are arranged in groups with the objective of enhancing the ultimate uplift load. With a pile spacing of 3.3 times the pile diameter (3.3*D*), the interaction of the soil between the piles is significantly more pronounced. Preliminary simulation verification revealed that when the enlargement factor exceeds 2.8, the soil between the piles becomes unstable due to excavation unloading. This is specifically manifested as soil collapse phenomena during the simulation process, leading to a drastic increase in computational cost and difficulty in achieving convergence. Therefore, combining engineering practice with simulation feasibility, the upper limit of the enlargement factor research range in this paper is set to 2.8, while the lower limit is set to 1, representing a straight pile foundation.

**Fig 16:**
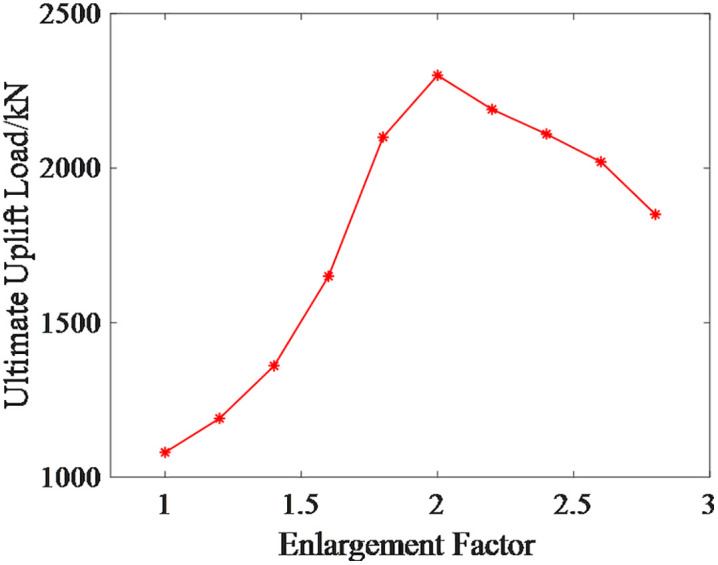
Ultimate Uplift Load Curve for Micro Under-reamed Pile at Different Enlargement Factors.

The relationship curve between the enlargement factor and the ultimate uplift load of the micro-enlarged diameter pile foundation is shown in Fig 16.From the results shown in Fig 16, it can be observed that the ultimate uplift load of the micro-enlarged diameter pile foundation increases and then decreases with the enlargement factor, reaching its maximum value around an enlargement factor of 2. The reason for this variation is that, in the initial stage of the curve, the primary resistance to the uplift load of the micro-enlarged diameter pile foundation shifts from friction between the pile body and the soil to friction between the enlarged diameter head and the soil. The increase in the enlargement factor significantly enhances the ultimate uplift load of the foundation.After reaching the peak of the curve, as the enlarged diameter head continues to increase, the amount of soil between the piles decreases and the pile group effect increases, leading to a reduction in the ultimate uplift load of the micro-enlarged diameter pile foundation.

**Fig 17:**
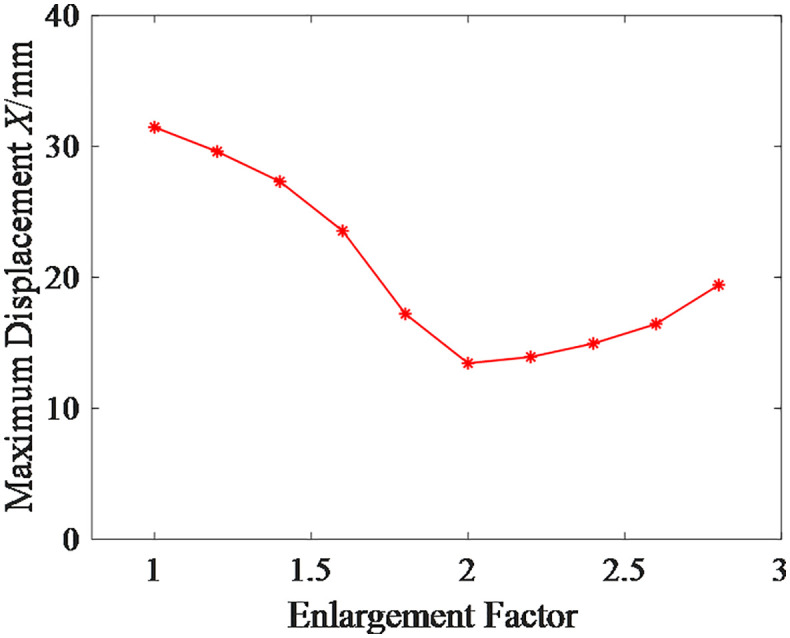
Maximum Displacement of Micro Under-reamed Pile at Different Enlargement Factors.

Using the same method, the maximum displacement of the micro-enlarged diameter pile foundation at different enlargement factors under the ultimate uplift load of the micro-straight pile foundation is shown in Fig 17.The corresponding risk values, risk reduction rates, and risk levels are shown in Table 6.From the results shown in Table 6, it can be observed that the risk reduction effect of the micro-enlarged diameter pile foundation is more significant when the enlargement factor is between 2 and 2.4.

**Table 5:** Physical and mechanical parameters of soil layers at the test site.

Sample No。	Sample Depth/m	Water Content/%	Compressibility Modulus/MPa	Cohesion/kPa	Internal Friction Angle/°
1−1	0 ~ 3.0	22.94	14.90	12.50	25.60
1-2	0 ~ 3.0	20.12	15.30	14.89	28.45
2−1	3.0 ~ 3.9	19.89	18.80	20.47	15.05
3−1	5.2 ~ 6.2	22.18	19.40	23.08	17.39
3−2	5.2 ~ 6.2	21.86	21.50	23.61	17.87
4−1	8.0 ~ 9.0	24.43	15.40	17.18	30.49

#### 4.2.3. Mechanical properties and risk assessment of micro-inclined pile foundation.

**Fig 18:**
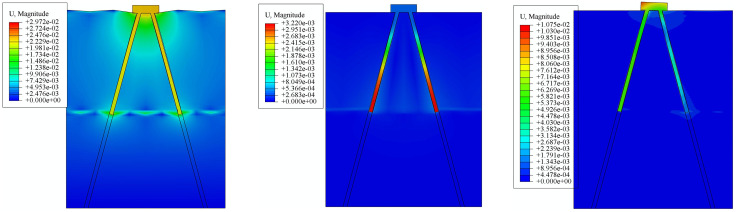
Displacement Simulation Results of Inclined Pile Foundation under Ultimate Loads. (a) Displacement under Ultimate Compressive Load. (b) Displacement under Ultimate Uplift Load. (c) Displacement under Ultimate Horizontal Load.

Based on the simulation model shown in Fig 5, the inclination angle of the micro-pile foundation’s pile body is changed (the inclination angle selected for this simulation is 15°), thereby establishing a pile-soil system mechanical property simulation model for the micro-inclined pile foundation.Through pile-soil system mechanical property simulation, the ultimate compressive load, ultimate uplift load, and ultimate horizontal load of the micro-inclined pile foundation are determined to be 2900kN, 1140kN, and 960kN, respectively.Compared to the micro-straight pile foundation, the three ultimate loads of the micro-inclined pile foundation are increased by 3.57%, 5.56%, and 54.84%, respectively. It can be observed that the micro-inclined pile mainly improves its resistance to horizontal loads.The displacement simulation results of the micro-inclined pile foundation under its ultimate loads are shown in Fig 18. Fig 18(a) shows the displacement under the ultimate compressive load, Fig 18(b) shows the displacement under the ultimate uplift load, and Fig 18(c) shows the displacement under the ultimate horizontal load (direction to the right).

**Fig 19:**
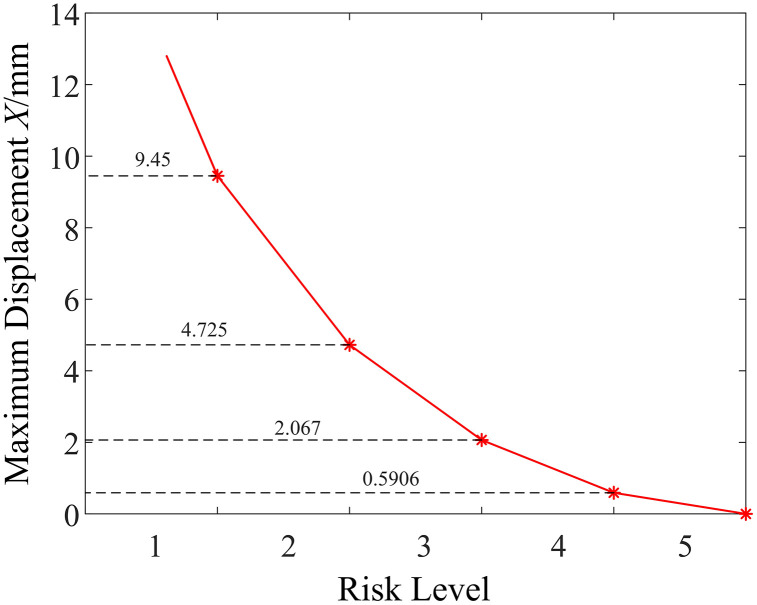
Maximum Displacement and Risk Level Relationship Curve of Micro-Inclined Pile Foundation.

From the results shown in Fig 18(a), it can be seen that under the ultimate compressive load, the displacement of the micro-inclined pile foundation and the deformation of the surrounding soil mainly occur around the cap and the pile body. The closer the soil is to the micro-inclined pile foundation, the larger the displacement, and the influence of the micro-inclined pile foundation on the soil extends further.Significant deformation of the soil around the cap and at the base of the inclined pile occurs due to the compressive load.As illustrated in Fig 18(b), the displacement of the micro-inclined pile foundation and the surrounding soil mainly occurs in the area vertically above the inclined pile shaft. This deformation pattern confirms the activation of the “soil wedge” mechanism. Unlike straight piles, the inclined geometry forces the pile to lift the overlying soil column during uplift. This interaction increases the normal stress acting on the pile-soil interface due to the weight of the soil wedge, thereby enhancing the frictional resistance. Furthermore, the deformation observed around the pile tip indicates that the deep soil layers are fully mobilized, contributing to the overall stability through the geometric interlocking effect. From the results shown in Fig 18(c), it can be seen that under the ultimate horizontal load (direction to the right), the deformation of the micro-inclined pile foundation with “uplift” on one side and “compression” on the other side shows differences compared to the micro-straight pile foundation.This is because the inclined pile on the side opposite to the direction of the applied force exhibits the characteristics of a negative inclined pile, while the inclined pile on the side in the same direction as the applied force exhibits the characteristics of a positive inclined pile, which greatly enhances the pile group’s resistance to horizontal loads.The soil around the pile tip on the side in the same direction as the applied force experiences greater deformation.Furthermore, the risk mitigation effect of the micro-inclined pile foundation is evaluated.Using the same approach as for the micro-enlarged diameter pile foundation, the ultimate horizontal load of the micro-straight pile foundation is applied to the micro-inclined pile foundation. Through pile-soil system mechanical property simulation, the maximum displacement of the micro-inclined pile foundation is found to be 4.62 mm.Based on the improved LEC method proposed in Section 2 of this paper, the risk value of the micro-inclined pile foundation is calculated to be 4.89. The relationship curve between maximum displacement and risk level for the micro-inclined pile foundation is shown in Fig 19.From Fig 19, it can be seen that the risk level of the micro-inclined pile foundation at this point is Level 2.Compared to the traditional micro-straight pile foundation, the risk value of the proposed micro-inclined pile foundation decreases by 51.10%, and the risk level decreases from Level 1 to Level 2, achieving risk mitigation.

Furthermore, the variation pattern of the ultimate horizontal load of micro-inclined pile foundations with respect to the inclination angle is analyzed. According to relevant research results [30], inclined piles exhibit engineering construction feasibility within an inclination range of up to 30°. In this paper, the inclination angle range for the micro-inclined pile foundation is established as 0° to 25°. The angle adjustment precision of existing small-scale drilling rigs for mountainous terrain, combined with current on-site construction techniques, can fully satisfy the operational requirements within this inclination range.

**Fig 20:**
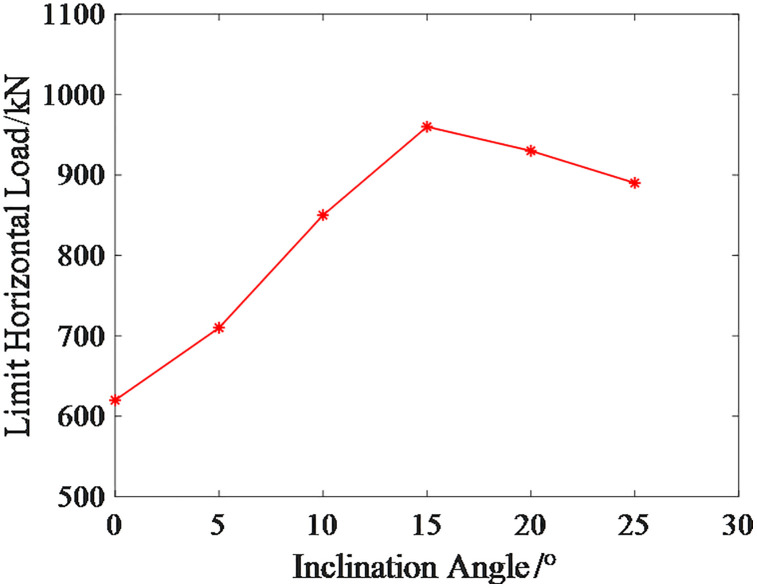
Limit Horizontal Load Curve of Micro-Inclined Pile Foundation at Different Inclination Angles.

The relationship curve between the inclination angle and the limit horizontal load of the micro-inclined pile foundation is shown in Fig 20. As observed from the results in Fig 20, the limit horizontal load of the micro-inclined pile foundation increases and then decreases as the inclination angle increases, with the maximum value occurring around an inclination angle of 15°.The reason for this variation is that, in the initial stage of the curve, the positive inclined pile on the side of the applied load direction exhibits end-bearing pile characteristics, while the negative inclined pile on the opposite side of the load direction is suppressed in its negative inclined pile characteristics. Since the increase in the positive inclined pile characteristics is greater than the suppression of the negative inclined pile characteristics, the increase in inclination angle significantly enhances the limit horizontal load. After reaching the peak of the curve, the increase in the positive inclined pile characteristics diminishes while the increase in the negative inclined pile characteristics becomes more pronounced, ultimately causing the limit horizontal load of the micro-inclined pile foundation to decrease.

**Fig 21:**
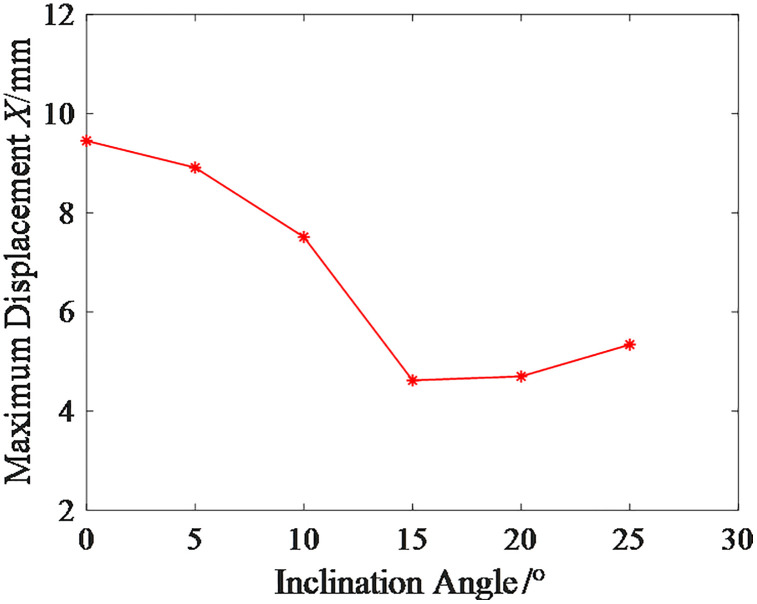
Maximum Displacement of Micro-Inclined Pile Foundation at Different Inclination Angles.

Using the same method, the maximum displacement of the micro-inclined pile foundation under the limit horizontal load of the micro-straight pile foundation at different inclination angles is obtained, as shown in Fig 21. The corresponding risk values, risk reduction rates, and risk levels are presented in Table 6. As seen from the results in Table 7, the risk reduction effect of the micro-inclined pile foundation is more significant when the inclination angle is between 15° and 20°.

**Table 6:** Risk Values and Risk Levels of Micro Under-reamed Pile at Different Enlargement Factors.

Enlargement Factor	1	1.2	1.4	1.6	1.8
Risk Value	10	9.41	8.68	7.48	5.47
Risk Reduction Rate/%	0	5.90	13.20	25.20	45.30
Risk Level	1	1	1	1	1
Enlargement Factor	2	2.2	2.4	2.6	2.8
Risk Value	4.27	4.42	4.75	5.22	6.17
Risk Reduction Rate/%	57.30	55.80	52.50	47.80	38.30
Risk Level	2	2	2	1	1

**Table 7:** Risk Values and Risk Levels of Micro-Inclined Pile Foundations at Different Inclination Angles.

Inclination Angle	0	5	10	15	20	25
Risk Value	10	9.43	7.95	4.89	4.97	5.65
Reduction Rate/%	0	5.70	20.50	51.10	50.30	43.50
Risk Level	1	1	1	2	2	1

## 5. Conclusion

1) Addressing the current lack of risk assessment methods for micro-pile foundations in mountainous transmission lines, this paper establishes a pile-soil system mechanical simulation model for micro-pile foundations. Based on the maximum displacement requirements during group pile foundation failure according to current standards, the ultimate load of the micro-pile foundation is calculated. The maximum displacement of the micro-pile foundation under ultimate load is correlated with the maximum value of the original LEC method risk factor in an equivalent ratio. This leads to the construction of a displacement-risk mathematical mapping for micro-pile foundations, thereby proposing an improved LEC method for risk assessment of micro-pile foundations.
2) This paper proposes structural measures for risk reduction of micro-pile foundations in mountainous transmission lines by using micro-diameter expansion pile foundations and micro-inclined pile foundations. The ultimate load of the micro-pile straight pile foundation is used as an excitation to simulate the bearing performance of the two improved micro-pile foundations. The maximum displacement of the two improved micro-pile foundations is calculated. Based on the proposed improved LEC method, the risk values and risk levels of both types of improved micro-pile foundations are computed and compared with those of traditional micro-straight pile foundations. The practical implementation of these measures requires rigorous quality control. For micro-inclined piles, modular lightweight drilling rigs adapted for mountainous terrain should be employed, with inclinometers used to ensure the drilling angle deviation remains within 2% and pile position tolerance within 50 mm. For micro-under-reamed piles, the enlargement factor is strictly limited to 2.8 based on this study’s stability analysis to avoid excessive disturbance to the inter-pile soil. Furthermore, mechanical caliper logging is recommended to verify the geometry of the enlarged bell, ensuring the actual anchorage dimensions meet design requirements.
3) The current risk assessment method utilizes a deterministic piecewise linear definition for the risk metric, primarily due to the scarcity of large-scale statistical data on pile foundation failures. Consequently, future research should focus on accumulating a broader database of field test cases to establish a rigorous probabilistic distribution for uplift failure. Additionally, the current study validates the method under specific geological conditions; further investigations involving diverse soil types (e.g., sandy soil or soft clay) and long-term cyclic loading are recommended to generalize the applicability of the proposed design perspective.

The results show that the risk values of the micro-diameter expansion pile foundation and the micro-inclined pile foundation are reduced, verifying the effectiveness of the proposed risk reduction measures.

## Supporting information

S1 Data Data for Fig 2. (XLSX)

S2 Data Data for Fig 11. (XLSX)

S3 Data Data for Fig 15. (XLSX)

S4 Data Data for Fig 16. (XLSX)

S5 Data Data for Fig 17. (XLSX)

S6 Data Data for Fig 19. (XLSX)

S7 Data Data for Fig 20. (XLSX)

S8 Data Data for Fig 21. (XLSX)
